# Forced Notch Signaling Inhibits Commissural Axon Outgrowth in the Developing Chick Central Nerve System

**DOI:** 10.1371/journal.pone.0014570

**Published:** 2011-01-21

**Authors:** Ming Shi, Zhirong Liu, Yonggang Lv, Minhua Zheng, Fang Du, Gang Zhao, Ying Huang, Jiayin Chen, Hua Han, Yuqiang Ding

**Affiliations:** 1 Department of Neurology, Xijing Hospital, Fourth Military Medical University, Xi'an, China; 2 Department of Vascular and Endocrine Surgery, First Affiliated Hospital, Fourth Military Medical University, Xi'an, China; 3 Department of Medical Genetics and Developmental Biology, Fourth Military Medical University, Xi'an, China; 4 Department of Anatomy and Neurobiology, Tongji University School of Medicine, Shanghai, China; Medical College of Georgia, United States of America

## Abstract

**Background:**

A collection of *in vitro* evidence has demonstrated that Notch signaling plays a key role in the growth of neurites in differentiated neurons. However, the effects of Notch signaling on axon outgrowth in an *in vivo* condition remain largely unknown.

**Methodology/Principal Findings:**

In this study, the neural tubes of HH10-11 chick embryos were *in ovo* electroporated with various Notch transgenes of activating or inhibiting Notch signaling, and then their effects on commissural axon outgrowth across the floor plate midline in the chick developing central nerve system were investigated. Our results showed that forced expression of Notch intracellular domain, constitutively active form of RBPJ, or full-length Hes1 in the rostral hindbrain, diencephalon and spinal cord at stage HH10-11 significantly inhibited commissural axon outgrowth. On the other hand, inhibition of Notch signaling by ectopically expressing a dominant-negative form of RBPJ promoted commissural axonal growth along the circumferential axis. Further results revealed that these Notch signaling-mediated axon outgrowth defects may be not due to the alteration of axon guidance since commissural axon marker TAG1 was present in the axons in floor plate midline, and also not result from the changes in cell fate determination of commissural neurons since the expression of postmitotic neuron marker Tuj1 and specific commissural markers TAG1 and Pax7 was unchanged.

**Conclusions/Significance:**

We first used an *in vivo* system to provide evidence that forced Notch signaling negatively regulates commissural axon outgrowth.

## Introduction

Communication between the two sides of the bilaterally symmetrical central nerve system (CNS) is mediated by commissural axons. During vertebrate CNS development, these axons initially grow circumferentially toward the ventral midline floor plate and after crossing the midline they abruptly change their trajectory to project longitudinally towards their targets [1], [2]. A variety of molecules present along the dorsoventral and rostrocaudal axes of the neural tube have been shown to promote commissural axon growth and guidance towards and across the ventral midline. These molecules include neurotrophins, cell adhesion molecules, chemoattractants and chemorepellents [2], [3], [4], [5], [6], [7], [8]. Additionally, Notch signaling is found be also involved in the control of neurite outgrowth of differentiated neurons [9], [10].

Notch proteins are single-pass transmembrane cell surface receptors. Upon ligand binding, Notch receptors undergo proteolytic cleavage, resulting in the release of the Notch intracellular domain (NICD) that then translocates into the nucleus [11], [12], [13]. In the nucleus, NICD binds to the transcription factor recombination signal binding protein-J (RBPJ) and activates the transcription of target genes, such as the hairy and enhancer of split (HES) homologues Hes1 and Hes5 [14].

The Notch pathway is most well-known for its crucial role in regulating cell fate decision during the development of the CNS [15], [16]. In addition to this canonical role, a group of *in vitro* studies have provided evidence showing that Notch pathway is also involved in modulating neurite growth in the differentiated neurons. In Drosophila, for example, Notch affects axonal extension by regulating the Abl kinase signaling pathway [17]. Notch signaling is also known to promote dendritic branching [9], and to inhibit neurite extension in cultured rodent cortical neurons [9], [10], [18], N2a neuroblastoma cells [19] and PC12 cells [20]. Furthermore, in cultured murine cortical neurons and differentiated human SH-SY5Y neuroblastoma cells, Notch signaling can induce microtubule stabilization in neurites and thereby promote neurite outgrowth and branching as well as growth cone enlargement [21], [22]. However, *in vivo* evidence demonstrating the effects of Notch signaling on axon outgrowth is still lacking.

Here we show that expressing transgenes that activate Notch signaling in the chick embryos at stage HH10-11 by *in ovo* electroporation has a profound effect on commissural axon outgrowth without affecting axon guidance and cell fate determination. Specifically we show that forced Notch signaling, with NICD, a constitutively active form of RBPJ (VP16), or a downstream transcriptional target of Notch, Hes1, significantly inhibited the growth of commissural axons across the floor plate midline, whereas inhibition of this pathway with a dominant-negative form of RBPJ (R218H) promoted circumferential outgrowth of commissural axons. These results provide first evidence in an *in vivo* system to show that Notch signaling activity negatively regulates axonal growth in the vertebrate CNS.

## Results

### Endogenous Notch signaling is present in chick commissural neurons

**Figure 1:**
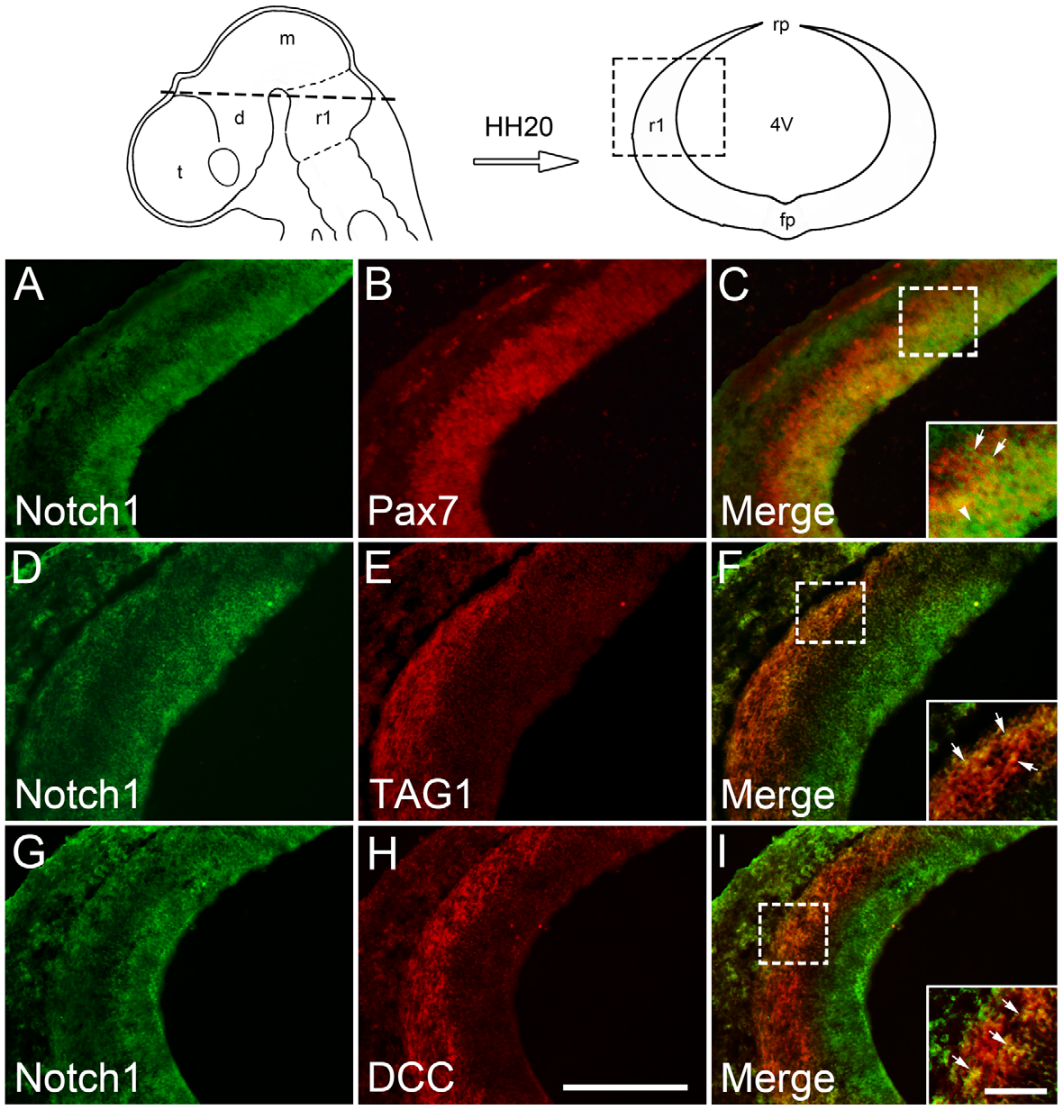
The expression of Notch1 in the commissural neurons. Double immunostaining of Notch1 (green) with Pax7 (red, A–C), TAG1 (red, D–F) and DCC (red, G–I) was performed on the sections from HH20 chick embryos to examine the expression of endogenous Notch signaling in the commissural precursors and neurons, respectively. The dashed line on the schematic diagram (top left) indicates the level of transverse sections. The dashed box on the diagram (top right) shows the region presented in (A–I). Insets in (C), (F) and (I) show the high magnification views of the boxed areas in their respective panels. Arrows in insets indicate the double-labeled cells and arrowhead shows the nuclear location of Notch1 immunoreactivity. 4V, fourth ventricle; d, diencephalon; fp, floor plate; m, mesencephalon; r1, rhombomere 1; rp, roof plate; t, telencephalon. Scale bars: 200 µm for (A–I); 50 µm for insets in (C), (F) and (I).

We first examined whether endogenous Notch signaling is present in the commissural neurons. Double immunostaining of Notch1 with commissural precursor marker Pax7 [23], [24] or commissural neuron markers TAG1 and DCC [25] was performed on the hindbrain sections from HH20 chick embryos. Our results showed that Notch1 was mainly expressed in the ventricular zone and colocalized well with Pax7 (Fig. 1A–C). Meanwhile, a weak immunoreactivity for Notch1 was also observed in the mantle zone where Notch1 expression partially overlapped with TAG1 (D–F) or DCC (G–I). Note that Notch1 immunostaining principally showed a cytoplasmic localization and nuclear staining was only observed in some cells (arrowhead in Fig. 1C). The colocalization of Pax7, TAG1 and DCC with Notch1 indicates the possible role of Notch1 signaling in the commissural neurons.

### Forced expression of Notch signaling inhibits commissural axon outgrowth

**Figure 2:**
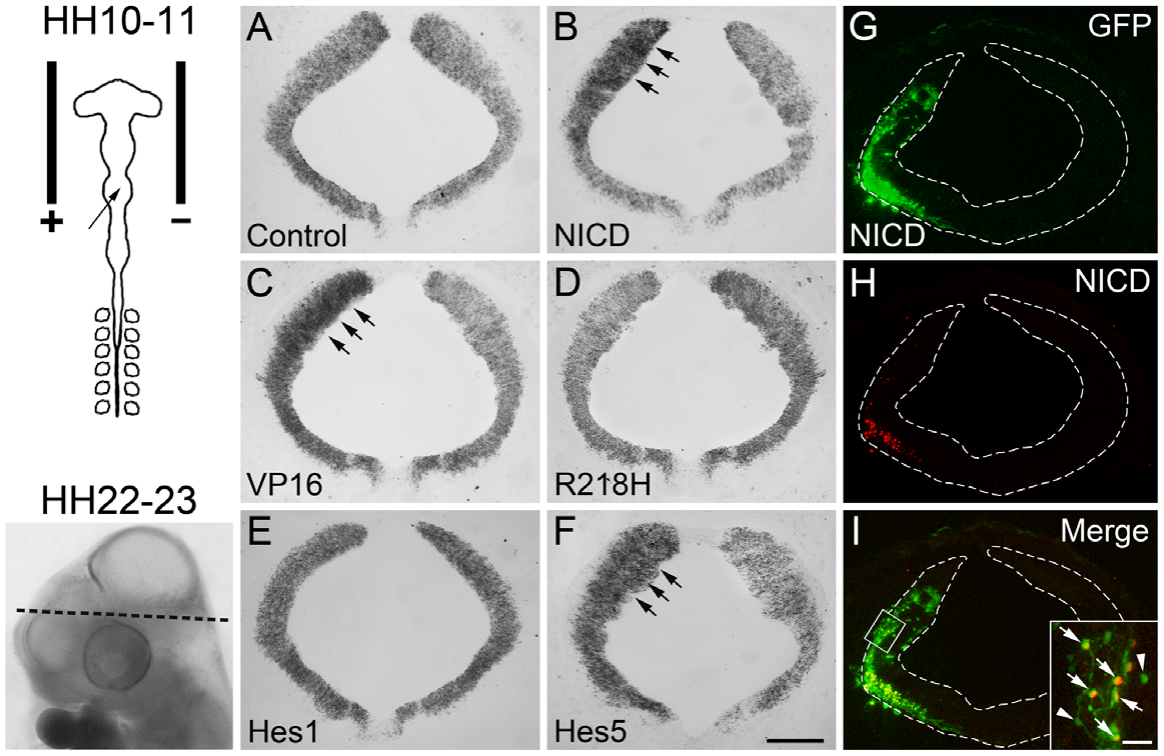
The expression of Notch target gene Hes5 and NICD in chick embryos after Notch transgene delivery. The rostral hindbrains of HH10-11 chick embryos were *in ovo* electroporated with the expression vectors indicated. The schematic diagram (Top left) depicts the electroporation procedure. The dashed line on the whole-mount embryo (bottom left) shows the level of transverse sections presented in (A–F). (A–F) HH10-11 hindbrains were electroporated and Hes5-hybridized at HH22-23. (A) In the control HH22-23 hindbrain electroporated with empty vector alone, Hes5 is expressed normally in the ventricular zone. Mis-expression of NICD (B), VP16 (C) or Hes5 (F) upregulates Hes5 expression on the electroporated side (left hemisphere, arrows), compared to that on the contralateral side (right hemisphere). (D) Expressing R218H slightly downregulates Hes5 expression while Hes1 expression (E) does not affect Hes5 expression on the electroporated side. (G–I) Chicken hindbrains were electroporated with NICD construct at HH10-11 and double-immunostained with GFP (G, green) and NICD (H, red) at HH22-23. Inset in (I) is the high magnification of the boxed areas. Arrows in insets refer to GFP/NICD double-positive cells and arrowheads refer to GFP single-positive cells. Scale bars: 200 µm for (A–I); 25 µm for the inset in (I).

To investigate the possible effects of Notch signaling on commissural neurons, we electroporated the rostral hindbrain of HH10-11 chick embryos with plasmid encoded EGFP alone or with bicistronic expressing plasmids containing NICD, VP16, R218H, Hes1 or Hes5 cDNAs. On the transverse sections from HH22-23 electroporated embryos at the level indicated by the dashed line in Figure 2, we first examined the efficiency of Notch transgenes after electroporation using *in situ* hybridization of Hes5, a target gene of Notch signaling. Our results showed that Hes5 expression was upregulated on the electroporated side after delivery of NICD, VP16 and Hes5 constructs, slightly downregulated after delivery of R218H construct, and unchanged after delivery of empty plasmid (Fig. 2A–F). These findings suggest that these plasmids worked well in our electroporation system. To assess transgene protein expression, we performed the double immunostaining of GFP with activated Notch1 (NICD) (Fig. 2G–I). Our results showed that NICD was highly expressed in most GFP-positive cells with a nuclear location. Note that NICD-immunoreactive signals were not observed in non-electroporated cells of chick hindbrain. This may be explained by the fact that NICD antibody used here (V1744) is specifically reactive to the rodent but not chick, thus not recognizing the endogenous chick NICD proteins.

**Figure 3:**
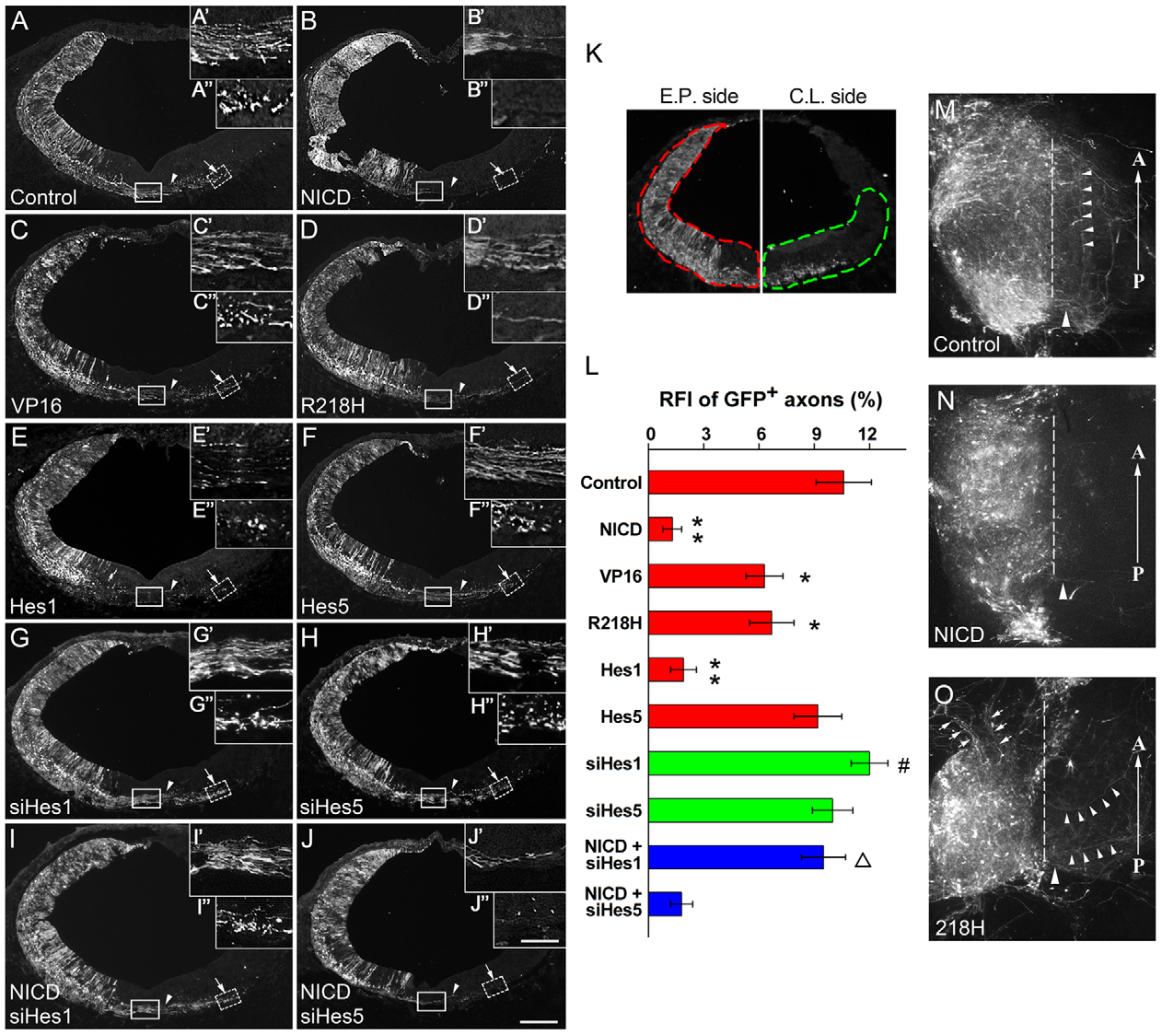
Modulation of Notch signaling affects axon outgrowth. (A–A'') A representative section from control embryos shows a large number of commissural axons crossing the floor plate midline in the rostral hindbrain. After growing circumferentially towards the ventromedial region, the axons turn and project along the longitudinal axis. (B–B'') Mis-expression of NICD results in a drastic decrease in the number of commissural axons crossing the midline. Only a very few axons are observed in the ventrolateral region of the contralateral rostral hindbrain. (C–C'') VP16 expression reduces the number of commissural axons midline crossing, and the projection patterns in the contralateral side do not appear affected. (D–D'') The expression of R218H seems also to lead to a reduction in the number of commissural axons crossing the midline while the axons that cross the midline maintain a linear, circumferential trajectory into the ventromedial region of the contralateral side. (E–E'') Hes1 mis-expression has a similar effect on axon outgrowth as that of NICD. (F–F'') Hes5 mis-expression does not affect commissural axon outgrowth. (G–G'') Hes1 knockdown by siRNA (siHes1) significantly increases the number of axons crossing midline and projecting along the circumferential number as compared with Hes1 mis-expression. (H–H'') Hes5 knockdown by siRNA (siHes5) seems not to affect axon outgrowth. (I–I'') Hes1 knockdown rescues the phenotype of axonal growth inhibition induced by NICD. (J–J'') Hes5 knockdown does not have effects on NICD-induced axonal phenotype. Arrowheads in all panels indicate axons crossing the midline, and arrows indicate axons extending in the longitudinal plane. All insets show the high magnification views of the boxed areas in their respective panels. Scale bars: 200 µm for (A–J); 50 µm for (A'–J', A''–J''). (K) The panel shows the method to normalize relative fluorescence intensity (RFI) of GFP^+^ axons. Red and green dash lines refer to the regions for quantitation in the electroporated (E.P.) and contralateral (C.L.) side, respectively. (L) Quantitation of GFP^+^ axons crossed to the contralateral hemisphere. *, *p*<0.05; **, *p*<0.01, compared to the control; #, *p*<0.05, compared to Hes1; **Δ**, *p*<0.05, compared to NICD. (M–O) GFP immunostaining was performed on whole-mount filets electroporated with empty vector (M), NICD (N) and 218H (O). In control filets (M), a number of GFP^+^ axons cross the midline and change their trajectories along the anterior-posterior (A–P) axis (small arrowheads). Forced expressing NICD (N) significantly reduces the number of GFP^+^ axons. Mis-expressing R218H (O) enhances GFP^+^ axons to grow on the electroplated side (arrows), and project alone circumferential (arrowheads), but not A–P axis. Dash lines indicate the midline in the floor plate. Large arrowheads refer to the axons across the midline.

Then GFP immunostaining was performed to detect the axons from electroporated cells. On the electroporated side of control embryos, a large number of GFP-labeled commissural axons were observed growing toward and crossing the ventral midline (Fig. 3A, A'). After crossing, axons continued to grow circumferentially through the contralateral ventromedial region (arrowhead in Fig. 3A) and then, in the ventrolateral region, the distal ends of labeled axons extended along the longitudinal axis (indicated by punctate GFP staining in transverse sections, Fig. 3A, A'', arrow). Forced expressing NICD in the rostral hindbrain at HH10-11 caused a striking change to commissural axon outgrowth. At HH22-23, very few labeled axons were observed growing toward the midline (Fig. 3B, B'), and consequentially very little GFP labeling was observed in the midline area, as well as the ventromedial and ventrolateral regions of the contralateral side (Fig. 3B, B'', arrowhead and arrow). Compared with control electroporated embryos (10.6±1.5%), the expression of NICD decreased the relative fluorescent intensity (RFI) of GFP^+^ axons (1.3±0.5%) in ventral region of the contralateral hemisphere by 87% (p<0.01, Fig. 3L). The effect of activated Notch on commissural axon outgrowth is likely mediated by canonical Notch signaling pathway, since ectopic expression of Hes1 also inhibited the outgrowth of commissural neurons, as only a few labeled axons were observed crossing the midline (Fig. 3E–E''). In addition, VP16, a constitutively active form of RBPJ, also reduced the number of labeled commissural axons that cross the midline, albeit to a lesser degree than NICD or Hes1, and did not appear to affect the projection of axons that had crossed (Fig. 3C–C''). Quantification of the RFI of GFP^+^ axons in the ventral compartment of the contralateral hemisphere revealed that Hes1 mis-expression had a similar effect as NICD, reducing GFP RFI (1.9±0.7%, p<0.01; Fig. 3L) by over 80% relative to controls whereas VP16 had a more modest, but still significant, effect (6.3±1.0%, p<0.05; Fig. 3L). Unexpectedly, expressing R218H, a dominant-negative form of RBPJ, in the rostral hindbrain at HH10-11 also reduced the number and RFI (6.7±1.2%, p<0.05) of labeled post-commissural axons in the contralateral ventral hindbrain (Fig. 3D, D', L). Interestingly, R218H expression seemingly promoted circumferential outgrowth, as revealed by the lack of punctate GFP staining in the ventrolateral region of the contralateral side (Fig. 3D, D'', arrow). The counting of punctate staining showed a decrease in number by over 75% compared to controls. In contrast to Hes1, forced expressing Hes5 did not noticeably affect commissural axon outgrowth (9.2±1.3%, p>0.05; Fig. 3F–F'', L).

To observe the changes in axon outgrowth more clearly, we prepared whole-mount filet of electroporated hindbrain by cutting the dorsal midline. In control filet, many GFP^+^ axons crossed the midline, some of which changed their trajectories along the anterior-posterior axis (Fig. 3M, small arrowheads) on the contralateral side. By contrast, NICD mis-expression significantly reduced the number of GFP^+^ axons in the midline relative to control filet (Fig. 3N). R218H expression seemingly promoted the axons to grow along the circumferential, but not anterior-posterior axis on the contralateral side (Fig. 3O, arrowheads). We noted that on the electroporated side, significant axon outgrowth was observed (arrows in Fig. 3O), indicating that inhibiting Notch signaling may actually promote axon outgrowth. However, these axons failed to project to the contralateral side and alternatively stayed on the electroporated side, implying a defect in axon guidance. To further clarify the effects of Notch signaling on axon outgrowth, we performed additional siRNA knockdown experiments. After knockdown of Hes1 by siRNA, commissural axons toward and across the midline was somewhat enhanced, though no significant difference was observed as compared with the control (Fig. 3G–G'', L). However, Hes1 knockdown significantly promoted commissural axons to grow alone the circumferential axis (Fig. 3G''). By contrast, Hes5 knockdown seemed to have no effect on commissural axon outgrowth (Fig. 3H–H'', L). Moreover, to determine whether the observed axon outgrowth phenotype is an artifact of Notch overexpression, we performed the co-electroporation assays with a mixture of plasmids (NICD+Hes1-siRNA or NICD+Hes5-siRNA). Our data showed that knockdown of Hes1 by siRNA can rescue the phenotype of axon outgrowth inhibition induced by NICD ectopic expression (Fig. 3I–I'', L) while Hes5 knockdown had no effect on this phenotype (Fig. 3J–J'', L).

To determine whether Notch activity can suppress commissural axon outgrowth in other parts of the brain, we electroporated the six plasmids into the diencephalon (Fig. S1) and spinal cord (data not shown) at HH10-11 and analyzed cross-sections at HH22-23. In both these regions, we observed similar effects as those seen in the hindbrain. Taken together, these data suggest that forced Notch signaling negatively regulates commissural axon outgrowth in the developing chick CNS.

### Forced expression of Notch signaling does not affect commissural axon guidance

**Figure 4:**
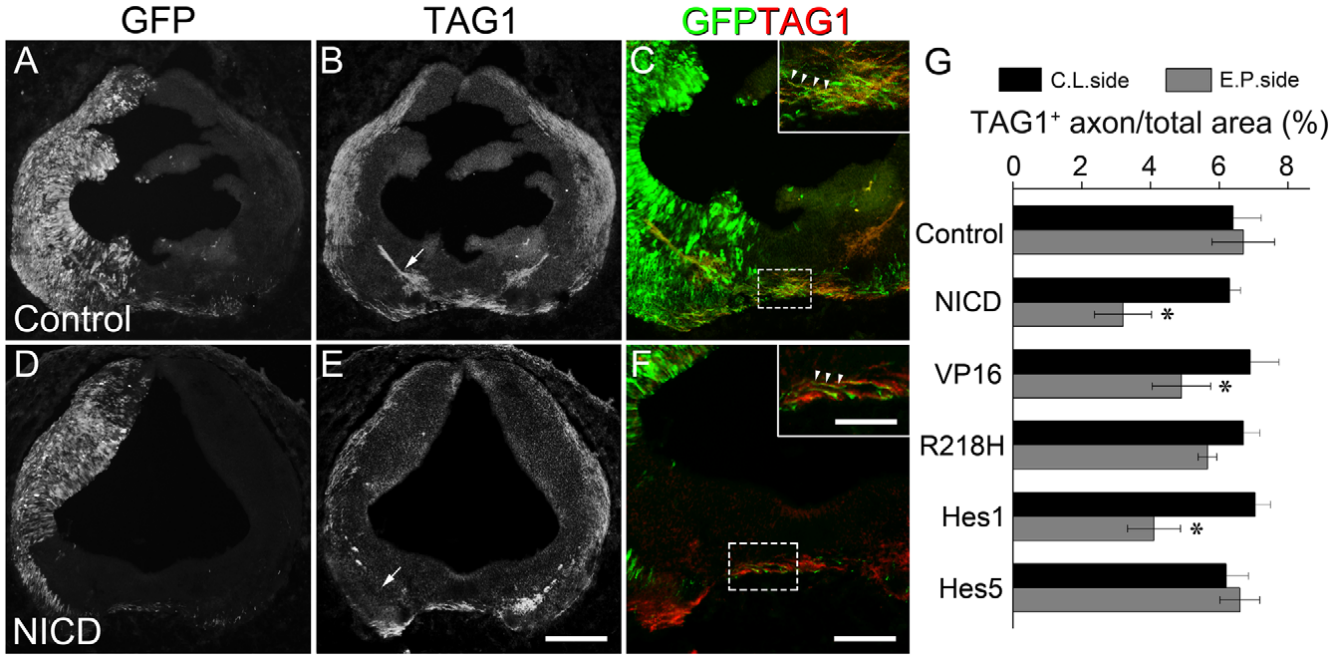
Forced Notch signaling does not affect commissural axons to cross the midline. Double immunostaining for GFP and TAG1 was performed after electroporation of empty and NICD vectors. (A–C) In the control, GFP^+^ (A) and TAG1^+^ (B) axons cross the ventral midline normally. GFP/TAG1 double-labeled axons are observed at the floor plate (C, arrowheads in inset). (D–F) NICD mis-expression decreases the number of GFP^+^ axons in the midline (D) and TAG1^+^ axons in the electroporated side (E, arrow) compared with those in the control (arrow in B). Although GFP^+^ axons toward across the midline are greatly reduced in number, the remainders are still GFP/TAG1 double-positive (F, arrowheads in inset). Insets in (C) and (F) show the high magnification views of the boxed areas in their respective panels. Scale bars: 200 µm for (A, B, D, E); 100 µm for (C, F); 50 µm for the insets in (C, F). (G) Quantitation of the ventral areas spanned by TAG1^+^ axons in the rostral hindbrain. Compared to those on the contralateral (C.L.) side, the expression of NICD, VP16 or Hes1 decreases the area ratios of TAG1^+^ axons on the electroporated (E.P.) side. R218H or Hes5 expression does not affect TAG1^+^ axons. *, *p*<0.05.

Axon guidance defect and delayed axon outgrowth may also account for the decrease in the number of axons across the midline observed above. To clarify these possibilities, we performed the immunostaining of TAG1 to mark the commissural axons after plasmid delivery. Electroporation of chick embryos with empty construct alone had no effect on the outgrowth of TAG1^+^ axons on the electroporated side, which behaved identically to those on the contralateral side (Fig. 4A–C, G, arrow in B). By contrast, the ventral areas spanned by TAG1^+^ axons were significantly reduced after mis-expression of NICD (Fig. 4D–G, arrows in E) compared with those on the contralateral side. Electroporated TAG1^+^ axons in the VP16 and Hes1, but not R218H and Hes5 embryos, showed a similar manner to those electroporated with NICD (Fig. 4G, and data not shown). Note that some GFP/TAG1 double-positive axons in the control and NICD embryos were observed in the ventral midline (Fig. 4C, F, arrowheads in inserts), indicating forced Notch activation may not affect axon guidance toward and across the midline. In addition, we also examined the changes in GFP^+^ axons at later embryonic stages (HH26-28), and found the similar inhibitory effects on commissural axon outgrowth as those at HH22-23 (data not shown), suggesting that axon outgrowth is inhibited rather than delayed after electroporation of Notch transgenes.

### Forced expression of Notch signaling does not affect cell fate determination of commissural neurons

**Figure 5:**
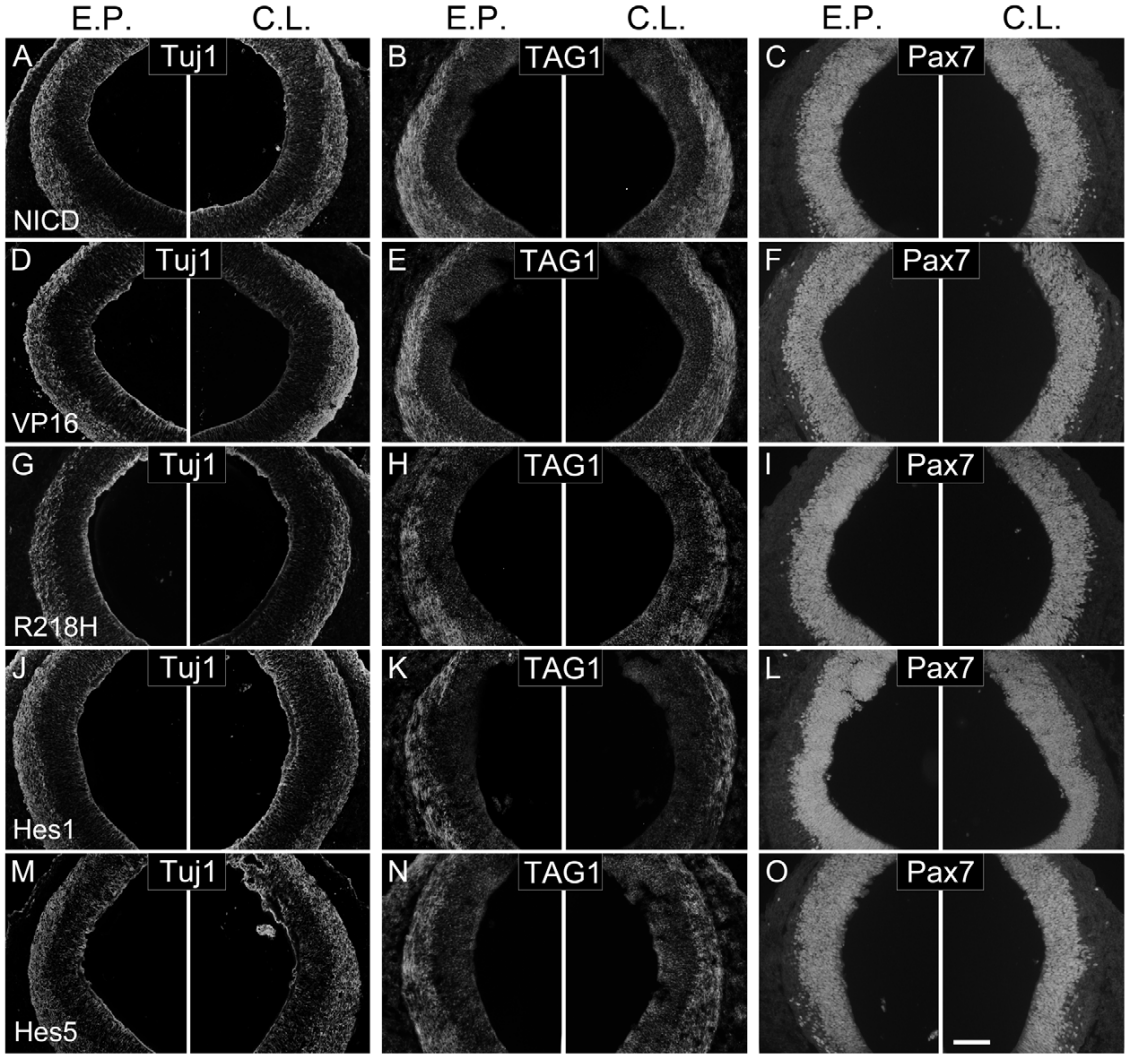
Modulation of Notch signaling does not affect cell fate determination of commissural neurons. After electroporation with the respective constructs, the transverse sections were immunostained with Tuj1 (A, D, G, J, M), TAG1 (B, E, H, K, N) or Pax7 (C, F, I, L, O). Mis-expression of NICD (A–C), VP16 (D–F), R218H (G–I), Hes1 (J–L) or Hes5 (M–O) appears not to affect the expression of Tuj1, TAG1 or Pax7 on the electroporated (C.L.) side, as compared to that on the contralateral (E.P.) side. Scale bar: 100 µm.

Notch signaling is well known for its roles in maintaining neural progenitor pools and inhibiting neuronal differentiation [16]. Thus, it is possible that inhibition of commissural axon outgrowth observed above is due to the changes in cell fate or the defects in differentiation of commissural neurons. To study this possibility, we performed immunostaining of postmitotic neuronal marker Tuj1 and commissural markers TAG1 and Pax7. Our results showed that the areas spanned by the Tuj1^+^, TAG1^+^ or Pax7^+^ cells on the electroporated side were comparative to those on the contralateral side (Fig. 5). In addition, we found that many GFP^+^ neurons from electroporated areas were able to complete their migration from the ventricular zone to the mantle zone (Figs. 2, 3, 4, Fig. S1), implying that they differentiated normally as commissural neurons. These data suggest that activating or repressing Notch signaling seems to have no effect on cell fate determination of commissural neurons.

## Discussion

The present study first uses an *in vivo* system to provide the evidence that Notch signaling regulates commissural axonal outgrowth in the developing chick CNS. Using *in ovo* electroporation, we ectopically expressed a number of constructs to activate or inhibit the Notch pathway in commissural neurons of the rostral hindbrain, diencephalon and spinal cord in chick embryos during periods of commissural axon outgrowth. Our data show that ectopically expressing NICD, VP16, or Hes1 all result in a significant decrease in the number of GFP^+^ axons toward and across the midline, whereas mis-expressing R218H and Hes1 knockdown promoted axon outgrowth along the circumferential axis. Although Notch signaling has profound effects on neuronal differentiation, we speculate that modulating Notch signaling did not affect cell fate determination of commissural neurons because the expression of postmitotic neurons marker Tuj1 and commissural markers TGA1 and Pax7 remained unaffected in the electroporated regions. Moreover, our results of TAG1 and GFP double staining revealed that forced Notch signaling seems not to affect the axon guidance to and across the ventral midline.

A group of studies over the past decade have provided *in vitro* evidence for a function of canonical Notch signaling in the regulation of neurite outgrowth in the nervous system [9], [10], [17], [18], [19], [22]; however, our study is the first to use an *in vivo* model to show that the canonical Notch pathway plays a vital role in axonal growth in the vertebrate nervous system. We found that ectopically expressing NICD in newly differentiated post-mitotic commissural neurons decreased axon midline crossing by about 87% compared to controls, indicating that activation of Notch signaling severely hinders axon outgrowth. This effect is likely dependent on the activation of Hes1, since mis-expression of RBPJ, a transcriptional activator for Hes1 [16], and of Hes1 itself had similar effects on axonal extension on the contralateral side. Moreover, knockdown of Hes1 by siRNA can produce an opposite effect of NICD or RBPJ over-expression to some extent, and rescue NICD-induced axonal growth inhibition. By contrast, Hes5, though known to act redundantly with Hes1 in specifying cell fates [26], was found not to involve in the inhibition of axon outgrowth by our forced expression and knockdown assays. These findings imply that it is Hes1, but not Hes5 that most likely participates in the regulation of axonal growth, possibly via a Notch canonical pathway.

In the present study, an unexpected finding is that the dominant-negative form of RBPJ (R218H) seems to also inhibit axon outgrowth across the midline to a degree. This is counter-intuitive and difficult to understand in the context of the other results presented herein. However, our whole-mount filet assay showed that R218H can promote axon outgrowth on the electroporated side before crossing (Fig. 3). That is to say, inhibiting Notch signaling actually enhances axon outgrowth but meanwhile makes them lose their guidance to the midline, consequently causing more axons lingering on the electroporated side and less axons crossing the midline. Moreover, the finding that some axons which crossed the midline grow alone circumferential axis for a long distance (Fig. 3) may be another evidence for axonal growth enhancement. In fact, a previous study on *Drosophila* has already showed that inactivation of Notch activity causes a defect in axon guidance [27]. Thus, outgrowth and guidance decisions of commissural axons may be dependent on a balance of Notch activation and inactivation. Consistent with this possible binary effect of Notch signaling, a previous study found that inhibiting and activating Notch signaling could promote and inhibit neurite extension, respectively [10], [20], suggesting that the level of Notch activity can dynamically regulate neurite outgrowth.

The mechanisms underlying Notch-mediated axon outgrowth inhibition are still largely unknown. A line of *in vivo* evidence has demonstrated that Notch target gene Hes1 can directly bind to the promoter of MAP2 which is a neuron-specific protein, involving to stabilize microtubules and critical for neurite outgrowth and dendrite development, and subsequently represses MAP2 transcription [28], [29]. Another study also revealed that Hes1 can negatively regulate intracellular signal transduction stimulated by the neural cell adhesion molecules which are crucial to neurite outgrowth [30]. Therefore, Notch signaling is likely to inhibit axonal growth via regulating the expression of microtubule- or matrix-associated proteins.

In summary, we first use an *in vivo* system to provide the evidence that Notch signaling negatively regulates commissural axon outgrowth, most likely via the activation of canonical transcription-dependent signaling pathways.

## Materials and Methods

### Expression vectors and siRNA design

Expression vectors of VP16 and R218H under the control of the CMV promoter were provided by the Riken BioResource Center DNA Bank and used with the permission of Dr. T. Honjo (Riken, Kyoto, Japan). The cDNAs encoding NICD, and full-length Hes1 and Hes5 were obtained by reverse transcription polymerase chain reaction (RT-PCR) from E12.5 mouse embryo mRNA, and then cloned into the pCAGGS-IRES-EGFP plasmid. Restriction digests and DNA sequencing confirmed the correct orientation and reading frames of the constructs. The constructs were purified using Endotoxin-Free Plasmid Maxiprep Kit (Qiagen, GmbH, Hilden, Germany) according to the manufacturer's instructions. All encoding sequences (including VP16, NICD, Hes1, etc.) were inserted in front of IRES-EGFP cassette. Thus, in these bicistronic vectors, due to the presence of IRES (internal ribosome entry site), two open reading frames (e.g. NICD/EGFP; Hes1/EGFP, etc.) can be translated from one mRNA and monitored in the same cells by virtue of expression of EGFP on the same transcript. Control embryos were electroporated with empty pCAGGS-IRES-EGFP vector alone.

For targeted silencing of Hes1 and Hes5 expression, Hes1 and Hes5 siRNAs were cloned into pSUPER.retro vector (Oligoengine, Seattle, WA, USA) according to the manufacturer's introduction. Hes1and Hes5 siRNA sequences were 5′- CGGCCAATTTGCCTTTCTC-3′, 5′-GCCCTGGGATTACAAGGAT-3′, respectively, predicted using the online software BLOCK-iT™ RNAi Designer available from Invitrogen (https://rnaidesigner.invitrogen.com). The resulting vectors were confirmed by restriction enzyme digestion and DNA sequencing. The interfering efficiency was examined by Western blot after co-transfection of respective expressing and siRNA constructs in HEK293 cells (data not shown).

### *In ovo* electroporation

Fertilized chicken eggs were incubated at 38°C under humid conditions for 40 h to stage HH10-11. Expression plasmids (0.5 µl of 1.0 µg/µl in sterile PBS) were injected into the fourth ventricle or the spinal neural tube with glass capillaries (see schematic in Fig. 2). After injection, platinum electrodes (Nepa Gene Co., Ltd, Chiba, Japan) were placed parallel to the neural tube with 4 mm distance between the anode and cathode. The embryos were pulsed 5 times (20 V for 50 ms) at 1 s intervals using an Electro Square Porator ECM830 (BTX, Holliston, MA, USA). For co-electroporation, NICD and Hes1-/Hes5-siRNA vectors in a 1∶1 ratio were mixed before injection into neural tube. Electroporated embryos were incubated for another 48 h to stage HH22-23, and then harvested for immunohistochemistry and *in situ* hybridization.

### Immunohistochemistry and *in situ* hybridization

After fixing whole HH22-23 embryos in 4% paraformaldehyde in PBS at 4°C overnight, chicken brains and spinal cords were dissected out and sectioned transversely into 12 µm thick slices. For immunofluorescence, the sections were washed three times in 0.01 M PBS, blocked in PBS containing 2% normal donkey serum and 0.3% Triton X-100 for 0.5 h, and then incubated with the primary antibodies overnight at 4°C. For double immunostaining, two antibodies were added at the same time. The following primary antibodies were used: goat anti-DCC (A-20) (1∶400; Santa Cruz Biotechnology, Santa Cruz, CA, USA), rabbit anti-GFP (1∶2000; Molecular Probes, Eugene, OR, USA), rabbit anti-cleaved Notch1 (NICD) (Val1744; 1∶200, Cell Signaling Technology, Danvers, MA, USA), goat anti-Notch1 (C-20) (1∶100; Santa Cruz Biotechnology), mouse anti-Pax7 (1∶200; DSHB, Iowa City, IA, USA), mouse anti-TAG1 (1∶10; DSHB), and mouse anti-β-III-tubulin (Tuj1) (1∶1000; Chemicon, Billerica, MA, USA). For NICD and Notch1 immunostaining, the microwave antigen retrieval was performed on the tissue sections. A negative control was done by omission of the primary antibody. Species-specific secondary antibodies conjugated to Cy2 or Cy3 (1∶1000; Jackson ImmunoResearch, West Grove, PA, USA) were used to detect primary antibodies. After 3 h incubation at room temperature, the fluorescent signals were visualized under a Nikon 80i or a Zeiss LSM 510 confocal microscope. *In situ* hybridization of cryostat sections was performed as previously described [31]. Antisense DIG-labeled RNA probes of Hes5 were generated by RT-PCR from total RNA isolated from HH22 chicken embryos with Trizol (Life Technologies, Rockville, MD, USA). PCR-amplified DNA fragments were cloned into the pGEM-T vector (Promega, Madison, WI, USA).

### Statistical analysis

For quantitation of GFP-labeled axons in the regions of the contralateral side, Cy2 relative fluorescent intensity (RFI) was measured with the minimum threshold pixel intensity set at 80 as previously described [32]. The values of GFP RFI on the contralateral (C.L.) side were divided by those of GFP RFI measured from GFP-labeled cell bodies on respective electroporated (E.P.) side for normalization (see Fig. 3I). Normalized RFI  =  (RFI in C.L. side)/(RFI in E.P. side) ×100%. For quantitation of TAG1^+^ commissural axons, relative immunoreactive areas were measured by deriving the ratio of the areas spanned by TAG1^+^ axons on the electroporated side or contralateral side to the total area of hindbrain as described previously [33]. The GFP RFI and areas occupied by TAG1^+^ axons were measured on acquired images using NIH image-J software. For the control and each of the five experimental groups, a minimum of 10 sections from each of at least six electroporated embryos were analyzed. To ensure consistency between samples, we chose the embryos with similar electroporated efficiency as far as possible, and the data were collected by the persons not involved in this project. All the data were analyzed using ANOVA and two-tailed Student's t-test to perform statistical analysis.

## Supporting Information

Figure S1 Modulation of Notch signaling affects commissural axon outgrowth in the diencephalon of chick embryos. HH10-11 diencephalons were electroporated and GFP-immunolabeled at HH22-23 as described in Fig. 3. (A) In the control HH22-23 diencephalon, commissural axons initially project circumferentially and cross the floor plate midline. They extend towards the ventromedial region of the contralateral side, then turn and continue growing along the longitudinal axis. Mis-expression of NICD (B), VP16 (C), R218H (D), or Hes1 (E) transgene significantly decreases the number of commissural axons projecting towards and crossing the midline. (F) By contrast, Hes5 has no effect on commissural axons in the diencephalon. In all panels, arrowheads indicate axons crossing the midline, and arrows indicate axons extending in the longitudinal plane. The dashed line in the inset of (A) shows the level of transverse sections presented in (A-F). 3V, third ventricle. Scale bar: 100μm.
(3.42 MB TIF)

## References

[1] Colamarino SA, Tessier-Lavigne M (1995). The role of the floor plate in axon guidance.. Annu Rev Neurosci.

[2] Murakami F, Shirasaki R (1997). Guidance of circumferentially growing axons by the floor plate in the vertebrate central nervous system.. Cell Tissue Res.

[3] Guthrie S (2004). Axon guidance: mice and men need Rig and Robo.. Curr Biol.

[4] Hansen SM, Berezin V, Bock E (2008). Signaling mechanisms of neurite outgrowth induced by the cell adhesion molecules NCAM and N-cadherin.. Cell Mol Life Sci.

[5] Salinas PC (2003). The morphogen sonic hedgehog collaborates with netrin-1 to guide axons in the spinal cord.. Trends Neurosci.

[6] Stoeckli ET, Landmesser LT (1998). Axon guidance at choice points.. Curr Opin Neurobiol.

[7] Tessier-Lavigne M, Goodman CS (1996). The molecular biology of axon guidance.. Science.

[8] Zhu Y, Guthrie S, Murakami F (2006). Ephrin A/EphA controls the rostral turning polarity of a lateral commissural tract in chick hindbrain.. Development.

[9] Redmond L, Oh SR, Hicks C, Weinmaster G, Ghosh A (2000). Nuclear Notch1 signaling and the regulation of dendritic development.. Nat Neurosci.

[10] Sestan N, Artavanis-Tsakonas S, Rakic P (1999). Contact-dependent inhibition of cortical neurite growth mediated by notch signaling.. Science.

[11] Hu C, Dievart A, Lupien M, Calvo E, Tremblay G (2006). Overexpression of activated murine Notch1 and Notch3 in transgenic mice blocks mammary gland development and induces mammary tumors.. Am J Pathol.

[12] Irvin DK, Nakano I, Paucar A, Kornblum HI (2004). Patterns of Jagged1, Jagged2, Delta-like 1 and Delta-like 3 expression during late embryonic and postnatal brain development suggest multiple functional roles in progenitors and differentiated cells.. J Neurosci Res.

[13] Stump G, Durrer A, Klein AL, Lutolf S, Suter U (2002). Notch1 and its ligands Delta-like and Jagged are expressed and active in distinct cell populations in the postnatal mouse brain.. Mech Dev.

[14] Kageyama R, Ohtsuka T (1999). The Notch-Hes pathway in mammalian neural development.. Cell Res.

[15] Gaiano N, Fishell G (2002). The role of notch in promoting glial and neural stem cell fates.. Annu Rev Neurosci.

[16] Louvi A, Artavanis-Tsakonas S (2006). Notch signalling in vertebrate neural development.. Nat Rev Neurosci.

[17] Giniger E (1998). A role for Abl in Notch signaling.. Neuron.

[18] Berezovska O, McLean P, Knowles R, Frosh M, Lu FM (1999). Notch1 inhibits neurite outgrowth in postmitotic primary neurons.. Neuroscience.

[19] Franklin JL, Berechid BE, Cutting FB, Presente A, Chambers CB (1999). Autonomous and non-autonomous regulation of mammalian neurite development by Notch1 and Delta1.. Curr Biol.

[20] Levy OA, Lah JJ, Levey AI (2002). Notch signaling inhibits PC12 cell neurite outgrowth via RBP-J-dependent and -independent mechanisms.. Dev Neurosci.

[21] Ferrari-Toninelli G, Bonini SA, Bettinsoli P, Uberti D, Memo M (2008). Microtubule stabilizing effect of notch activation in primary cortical neurons.. Neuroscience.

[22] Ferrari-Toninelli G, Bonini SA, Uberti D, Napolitano F, Stante M (2009). Notch activation induces neurite remodeling and functional modifications in SH-SY5Y neuronal cells.. Dev Neurobiol.

[23] Mansouri A, Gruss P (1998). Pax3 and Pax7 are expressed in commissural neurons and restrict ventral neuronal identity in the spinal cord.. Mech Dev.

[24] Otto A, Schmidt C, Patel K (2006). Pax3 and Pax7 expression and regulation in the avian embryo.. Anat Embryol (Berl).

[25] Saba R, Nakatsuji N, Saito T (2003). Mammalian BarH1 confers commissural neuron identity on dorsal cells in the spinal cord.. J Neurosci.

[26] Ohtsuka T, Ishibashi M, Gradwohl G, Nakanishi S, Guillemot F (1999). Hes1 and Hes5 as notch effectors in mammalian neuronal differentiation.. EMBO J.

[27] Crowner D, Le Gall M, Gates MA, Giniger E (2003). Notch steers Drosophila ISNb motor axons by regulating the Abl signaling pathway.. Curr Biol.

[28] Maddodi N, Bhat KM, Devi S, Zhang SC, Setaluri V (2010). Oncogenic BRAFV600E induces expression of neuronal differentiation marker MAP2 in melanoma cells by promoter demethylation and down-regulation of transcription repressor HES1.. J Biol Chem.

[29] Bhat KM, Maddodi N, Shashikant C, Setaluri V (2006). Transcriptional regulation of human MAP2 gene in melanoma: role of neuronal bHLH factors and Notch1 signaling.. Nucleic Acids Res.

[30] Jessen U, Novitskaya V, Walmod PS, Berezin V, Bock E (2003). Neural cell adhesion molecule-mediated neurite outgrowth is repressed by overexpression of HES-1.. J Neurosci Res.

[31] Shi M, Guo C, Dai JX, Ding YQ (2008). DCC is required for the tangential migration of noradrenergic neurons in locus coeruleus of mouse brain.. Mol Cell Neurosci.

[32] Wang CL, Zhang L, Zhou Y, Zhou J, Yang XJ (2007). Activity-dependent development of callosal projections in the somatosensory cortex.. J Neurosci.

[33] Okada A, Charron F, Morin S, Shin DS, Wong K (2006). Boc is a receptor for sonic hedgehog in the guidance of commissural axons.. Nature.

