# Supplementary figures and images for: Forced Notch Signaling Inhibits Commissural Axon Outgrowth in the Developing Chick Central Nerve System
Source: PLoS One. 2011 Jan 21;6(1):e14570. doi: 10.1371/journal.pone.0014570 (PMC3024975; doi:10.1371/journal.pone.0014570)

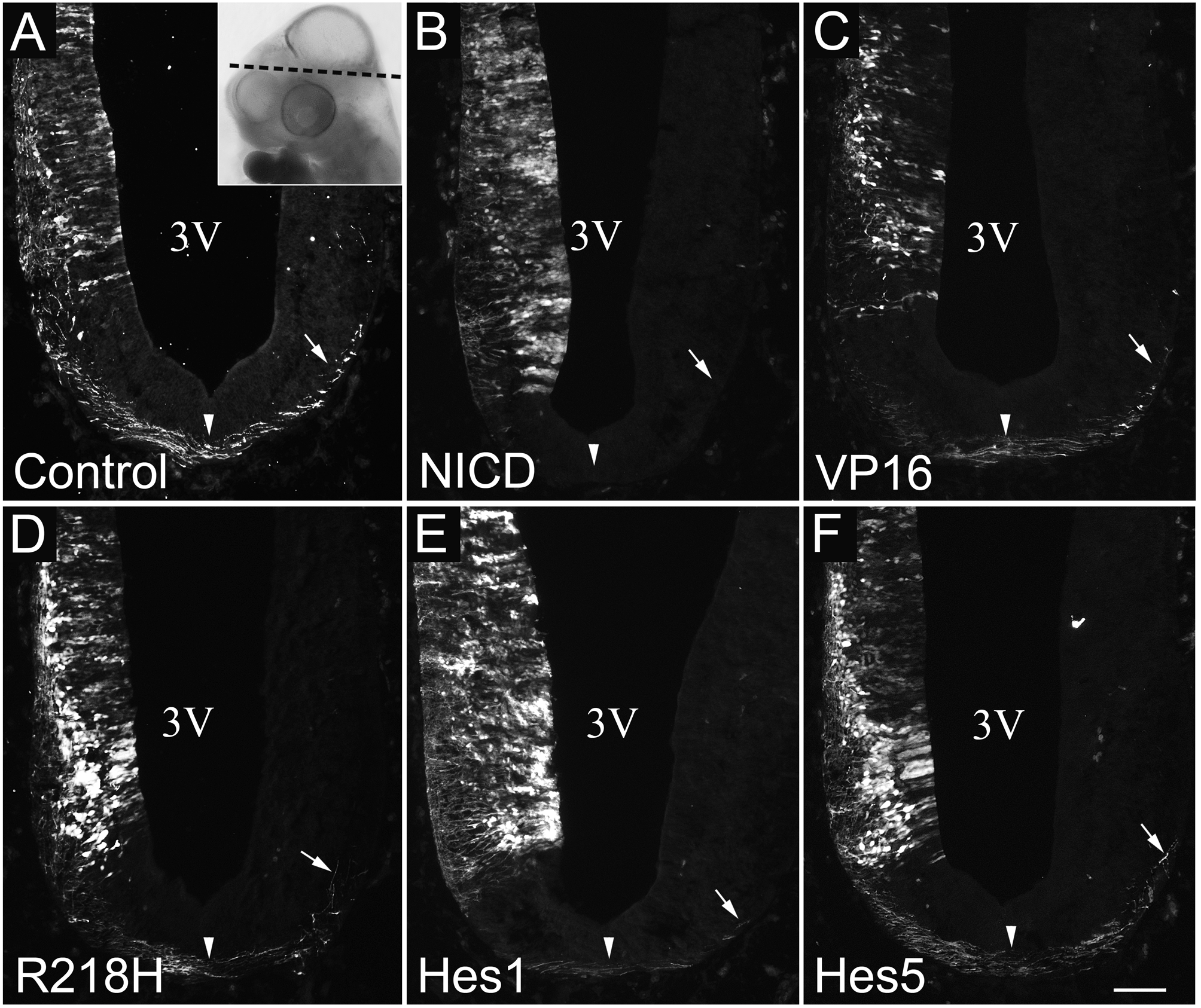

Supplement: Figure S1 — Modulation of Notch signaling affects commissural axon outgrowth in the diencephalon of chick embryos. HH10-11 diencephalons were electroporated and GFP-immunolabeled at HH22-23 as described in Fig. 3. (A) In the control HH22-23 diencephalon, commissural axons initially project circumferentially and cross the floor plate midline. They extend towards the ventromedial region of the contralateral side, then turn and continue growing along the longitudinal axis. Mis-expression of NICD (B), VP16 (C), R218H (D), or Hes1 (E) transgene significantly decreases the number of commissural axons projecting towards and crossing the midline. (F) By contrast, Hes5 has no effect on commissural axons in the diencephalon. In all panels, arrowheads indicate axons crossing the midline, and arrows indicate axons extending in the longitudinal plane. The dashed line in the inset of (A) shows the level of transverse sections presented in (A-F). 3V, third ventricle. Scale bar: 100μm. (3.42 MB TIF) [file pone.0014570.s001.tif]
